# Mutation location of HCM-causing troponin T mutations defines the degree of myofilament dysfunction in human cardiomyocytes

**DOI:** 10.1016/j.yjmcc.2020.10.006

**Published:** 2020-10-24

**Authors:** Maike Schuldt, Jamie R. Johnston, Huan He, Roy Huurman, Jiayi Pei, Magdalena Harakalova, Corrado Poggesi, Michelle Michels, Diederik W.D. Kuster, Jose R. Pinto, Jolanda van der Velden

**Affiliations:** a Amsterdam UMC, Vrije Universiteit Amsterdam, Department of Physiology, Amsterdam Cardiovascular Sciences, Amsterdam, The Netherlands; b Department of Biomedical Sciences, College of Medicine, Florida State University, Tallahassee, FL, USA; c Institute of Molecular Biophysics, Florida State University, Tallahassee, FL, USA; d Department of Cardiology, Thorax Center, Erasmus Medical Center, Rotterdam, The Netherlands; e Department of Cardiology, Division Heart and Lungs, University Medical Center Utrecht, Utrecht, The Netherlands; f Regenerative Medicine Utrecht, University Medical Center Utrecht, Utrecht, The Netherlands; g Department of Experimental and Clinical Medicine, University of Florence, Florence, Italy

**Keywords:** Cardiomyopathy, Hypertrophic cardiomyopathy, Troponin, Troponin T, TNNT2, Myofilament, Mutation, Mutation location, Mutant protein dose, Human tissue, Troponin exchange, Protein level, Ca^2+^-sensitivity

## Abstract

**Background:**

The clinical outcome of hypertrophic cardiomyopathy patients is not only determined by the disease-causing mutation but influenced by a variety of disease modifiers. Here, we defined the role of the mutation location and the mutant protein dose of the troponin T mutations I79N, R94C and R278C.

**Methods and results:**

We determined myofilament function after troponin exchange in permeabilized single human cardiomyocytes as well as in cardiac patient samples harboring the R278C mutation. Notably, we found that a small dose of mutant protein is sufficient for the maximal effect on myofilament Ca^2+^-sensitivity for the I79N and R94C mutation while the mutation location determines the magnitude of this effect. While incorporation of I79N and R94C increased myofilament Ca^2+^-sensitivity, incorporation of R278C increased Ca^2+^-sensitivity at low and intermediate dose, while it decreased Ca^2+^-sensitivity at high dose. All three cTnT mutants showed reduced thin filament binding affinity, which coincided with a relatively low maximal exchange (50.5 ± 5.2%) of mutant troponin complex in cardiomyocytes. In accordance, 32.2 ± 4.0% mutant R278C was found in two patient samples which showed 50.0 ± 3.7% mutant mRNA. In accordance with studies that showed clinical variability in patients with the exact same mutation, we observed variability on the functional single cell level in patients with the R278C mutation. These differences in myofilament properties could not be explained by differences in the amount of mutant protein.

**Conclusions:**

Using troponin exchange in single human cardiomyocytes, we show that *TNNT2* mutation-induced changes in myofilament Ca^2+^-sensitivity depend on mutation location, while all mutants show reduced thin filament binding affinity. The specific mutation-effect observed for R278C could not be translated to myofilament function of cardiomyocytes from patients, and is most likely explained by other (post)-translational troponin modifications. Overall, our studies illustrate that mutation location underlies variability in myofilament Ca^2+^-sensitivity, while only the R278C mutation shows a highly dose-dependent effect on myofilament function.

## 1. Introduction

Hypertrophic cardiomyopathy (HCM) is the most prevalent inherited heart disease affecting 1:500 to 1:200 individuals in the general population [1,2]. Current genetic screening identifies a pathogenic gene variant (further referred to as mutation) ~50–60% of all patients [3]. Despite improved genotyping, prediction of disease based on genotype is challenging as the HCM population shows large clinical variability. This is evident from large differences in disease onset and severity, even in individuals carrying the exact same mutation. There is thus no clear genotype-phenotype relation as shown for mutations in the 3 most frequent affected sarcomere genes [4]. As the majority of genotype-positive individuals are heterozygous for a gene mutation, carrying one normal and one mutant allele, clinical heterogeneity may be explained by the abundance (dose) of the mutant protein. Although 50% of healthy and 50% of mutant protein would be expected with a heterozygous mutation, this ratio can change due to allelic expression being stochastic, which can result in variable expression levels of healthy and mutant protein [5,6]. Accordingly, 43% of mutant protein has been shown in human induced pluripotent stem cell-derived cardiomyocytes carrying the I79N mutation in the gene (*TNNT2*) encoding cardiac troponin T (cTnT) [7], whereas heterozygous *Tnnc1*-A8V mice only showed 21% of mutant protein [8]. Furthermore, mutant protein dose can be influenced by changes in protein stability and/or degradation. A mutant protein dose-dependent increase in myofilament Ca^2+^-sensitivity was reported in a transgene (Tg) mouse model with a α-tropomyosin mutation [9]. Another study showed a dose-dependent effect of the *Tnnt2* mutation R92Q on morphological and structural abnormalities as well as hypertrophy markers in Tg mice [10].

In addition to mutant protein dose, location of the mutation in the gene may explain the degree of cardiac muscle dysfunction. A mutation location effect has been observed in a study comparing two mouse models with different *Tnnt2* mutations, a missense and a truncation mutation, that differ in their degree of hypertrophy and fibrosis development [11]. Furthermore, studies in transgenic mice found that the *Tnnt2* mutation I79N increased Ca^2+^-sensitivity [12], whereas the R278C mutation did not [13]. Differences in Ca^2+^-sensitivity and Ca^2+^-binding affinity have also been demonstrated in a study comparing the mouse models for *Tnnt2* I79N, F110I and R278C [14]. Similarly, in vitro studies using recombinant proteins have shown that the HCM-associated increase in Ca^2+^-sensitivity differs for different human *TNNT2* mutations incorporated into porcine fibers [15], strengthening the concept of mutation location as a disease modifier.

Based on these previous studies, we hypothesize that the degree of myofilament dysfunction depends on both the mutant protein dose and mutation location. To test our hypothesis we make use of the troponin exchange method in human cardiomyocytes, which enables us to control the dose of mutant protein in single cardiac muscle cells and study the effects on myofilament Ca^2+^-sensitivity. We compared three pathogenic *TNNT2* mutations I79N, R94C and R278C. Furthermore, we were able to characterize myofilament function in three human myectomy samples of patients carrying the R278C mutation, which enabled us to compare the effects of this specific mutation in the absence (exchange experiments) and presence (myectomy samples) of secondary disease remodeling.

## 2. Methods

### 2.1. Human cardiac samples

Exchange experiments were performed in the human sample 2.114 which had a high endogenous phosphorylation background and was obtained from the Sydney Heart Bank. HCM tissue samples from the interventricular septum of patients harboring the *TNNT2* R278C mutation were obtained during myectomy surgery to relieve LV outflow tract obstruction and collected by the Erasmus University Medical Center Rotterdam and the University of Florence. HCM samples from patients with other mutations and without a sarcomere mutation (sarcomere mutation-negative) were used as controls in our protein analyses studies (samples from Erasmus Medical Center and Sydney Heart Bank). Table 1 provides an overview, including clinical characteristics, of all HCM samples used in this study. The samples 4.021, 5.033, 6.034, 7.012 and 7.040 were used as non-failing controls for single cardiomyocyte measurements and/or western blot analysis. They originate from the LV free wall of donor hearts without history of cardiac disease and were obtained from the Sydney Heart Bank. Written informed consent was obtained from each patient prior to myectomy and the study protocol was approved by the local medical ethics review committees. All samples were stored in liquid nitrogen until use.

### 2.2. Cardiomyocyte force measurements

Single cardiomyocytes were mechanically isolated from frozen cardiac tissue and functional myofilament measurements performed as described previously [16]. Briefly, the membrane of isolated cardiomyocytes was permeabilized with 0.5% Triton X-100 and single cardiomyocytes were attached to a force transducer and a motor needle. To determine the force-calcium relation, the force development of the cell was measured at different calcium concentrations. Ca^2+^-sensitivity of myofilaments was determined as the [Ca^2+^] needed to achieve half-maximal force (EC_50_). The protocol was performed at 1.8 and 2.2 μm sarcomere length of the cell to determine length-dependent activation (LDA), which was measured as the difference in EC_50_ at both sarcomere lengths. Only cells with a maximal force >10 kN/m^2^ were included in the analysis to ensure good quality of the cells.

### 2.3. Troponin exchange in cardiomyocytes

**Fig. 1.**
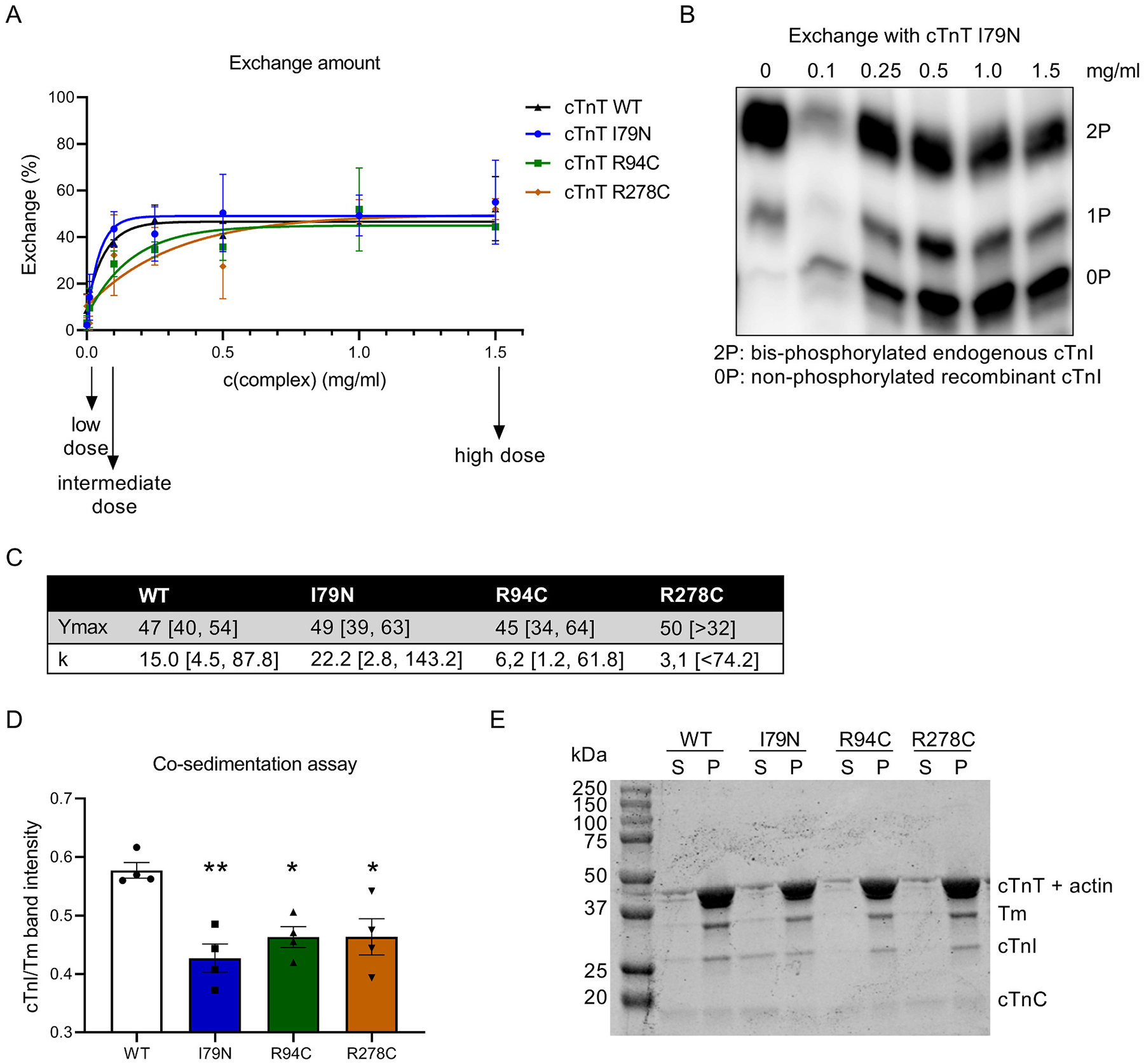
(A) Exchange of endogenous troponin complex with different concentrations of recombinant WT and mutant complex harboring the mutations I79N, R94C and R278C. Exchange percentage was analyzed by phos-tag gel analysis. (B) Representative image of phos-tag gel analysis for exchange curves in (A). (C) Y_max_ and k represent parameters of the exponential plateau curve fit with the 95% confidence interval (please note that an upper limit of Ymax and a lower limit of k for R278C could not be determined), *n* = 2. (D-E) Co-sedimentation assay. (D) Quantification of thin filament co-sedimentation assay results. Vertical axis is the band intensity ratio of cardiac troponin I to tropomyosin. (E) Representative image of an SDS-PAGE gel (12%) containing thin filament co-sedimentation products for WT and mutant troponin complexes. Abbreviations: S, supernatant; P, pellet; WT, wild-type; Tm, tropomyosin; cTnT, cardiac troponin T; cTnI, cardiac troponin I; cTnC, cardiac troponin C; one-way ANOVA with Dunnett’s multiple comparisons test, *p < 0.05, **p < 0.01 compared to WT. *n* = 4 for each condition.

Troponin exchange in permeabilized cardiomyocytes was performed as described previously [17]. The recombinant troponin complex was expressed and assembled as reported before [18]. The membrane-permeabilized cells of a highly phosphorylated human sample were incubated with non-phosphorylated recombinant troponin complex at the concentrations 0.01, 0.1 and 1.5 mg/ml, resembling a low, intermediate and high mutant protein concentration, respectively. Functional measurements after exchange were performed after incubation with protein kinase A (PKA) because the incorporated recombinant troponin complex was non-phosphorylated. The amount of troponin replacement was determined by phos-tag analysis of cardiac troponin I (cTnI) using the phosphorylation difference between the endogenous and the recombinant troponin complex, the former being highly phosphorylated and the latter being non-phosphorylated (example shown in Fig. 1B for the *TNNT2* I79N mutation). The percentage of troponin replacement after exchange was quantified as the percentage of non-phosphorylated cardiac troponin I (cTnI) of the total non-, mono- and bis-phosphorylated cTnI levels.

### 2.4. Co-sedimentation assay

Thin filament co-sedimentation assays were carried out using rabbit skeletal muscle filamentous actin (F-actin), recombinant human tropomyosin (Tm), and recombinant human troponin complexes as previously described [19,20] with a few modifications. Recombinant human cardiac tropomyosin was expressed with the N-terminal tag alanine-serine after the initiatior methione to mimic N-terminal acetylation [21]. Prior to performing the assay, Tm and troponin complexes were centrifuged to remove potential precipitates and the resultant supernatants were resolved on a denaturing gel to assess purity. Protein concentrations were determined using Pierce Coomassie Plus/Bradford assay (Thermo Fisher Scientific). F-actin, Tm, and troponin complexes were combined at a molar ratio of 7:1:1, respectively, into a final volume of 100 μl cosed buffer (25 mM HEPES, 60 mM NaCl, 3 mM MgCl2, 0.5 mM EGTA, 1 mM DTT, 0.5 mM CaCl2, 2 mM β-mercaptoethanol, pH 7.0) resulting in a final concentration of 20 μmol/l F-actin, 2.86 μmol/l Tm and 2.86 μmol/l troponin complex. The samples were centrifuged for 30 min at 120,000 × g in a TLA-100 rotor (Beckman Coulter) at 21 °C. The pellets (P) and supernatants (S) were subsequently diluted with reducing Laemmli buffer, boiled, and resolved on 12% SDS-PAGE gels. The proteins were stained with Coomassie, destained, and imaged on an Odyssey IR system (LI-COR Biosciences) followed by densitometric quantification using Image Studio V5.2 (LI-COR Biosciences).

### 2.5. Phosphorylation assays

Tissue samples were homogenized to a concentration of 2.5 μg/μl, and 100 μl of the homogenate was incubated with 10 units of protein kinase A (PKA; Sigma, P5511) and 0.006 mM cAMP for 40 min at 25 °C. The phosphorylation level before and after PKA incubation was assessed with ProQ phosphostaining and phos-tag gel analysis of cTnI. Troponin complexes were incubated with 0.5 units of PKA/μg and incubated at room temperature for 1, 5, 10, 20, 40 and 60 min. The phosphorylation level of cTnI was determined with phos-tag gel analysis.

### 2.6. Protein analysis

#### 2.6.1. Protein phosphorylation

Non-, mono- and bis-phosphorylated forms of cTnI (Pierce, MA1-22700) were quantified by phos-tag gel analysis as described previously [22]. It was used to assess the percentage of troponin replacement after exchange and to assess the cTnI phosphorylation level of human cardiac tissue samples.

#### 2.6.2. Protein levels

Whole tissue lysates were prepared to determine troponin protein levels. Therefore, pulverized frozen tissue was homogenized in 40 μl/mg tissue 1× reducing sample buffer (106 mM Tris-HCl, 141 mM Tris-base, 2% lithium dodecyl sulfate (LDS), 10% glycerol, 0.51 mM EDTA, 0.22 mM SERVA Blue G250, 0.18 mM Phenol Red, 100 mM DTT) using a glass tissue grinder. Proteins were denatured by heating to 99 °C for 5 min and debris was removed by centrifugation at maximum speed for 10 min in a microcentrifuge (Sigma, 1–15 K).

For analysis of troponin protein levels by Western blot, 5 μg of protein were separated on a 4–15% TGX gradient-gel (Biorad) and transferred to a polyvinylidene difluoride membrane. Site-specific antibodies directed to cTnT (ab10214, Abcam), cTnT (T6277, Sigma-Aldrich), cTnT (ab8295, Abcam), cTnI (ab10231, Abcam), cardiac Troponin C (cTnC, sc48347, Santa Cruz) and α-actinin (A7811, Sigma-Aldrich) were used to detect the proteins which were visualized with an enhanced chemiluminescence detection kit (Amersham) and scanned with Amersham Imager 600. Protein levels were determined by densitometric analysis. Protein levels were normalized to α-actinin or cTnI when appropriate.

Equal loading of troponin complexes was verified with Imperial protein stain (Thermo Scientific).

#### 2.6.3. Mass spectrometry to determine mutant protein dose in human tissue samples

Whole tissue lysates were run on 13% SDS gels to achieve a good separation of the cTnT and the actin band. Thereto approximately 15 μg of protein were loaded for each sample. The gel was stained with coomassie to visualize the protein bands and the cTnT band was cut out of the gel.

Subsequently, a wash buffer (50% aqueous acetonitrile with 50 mM ammonium bicarbonate) was used to de-stain cut gel bands. De-stained gel bands were then cut into ~1 mm pieces and shrunk by incubation with acetonitrile at 37 °C for 10 min. Gel pieces were further dried in SpeedVac (Thermo Fisher Scientific, Waltham, MA). A digestion buffer (10% aqueous acetonitrile with 50 mM ammonium bicarbonate) was added to rehydrate the gel pieces. Reductive alkylation of cysteine was carried out by mixing with 1,4-dithiothreitol (DTT, Sigma-Aldrich, catalog #: 11583786001, St. Louis, MO) at 37 °C for 10 min followed by mixing with iodoacetamide (IAA, Sigma-Aldrich, catalog #: I1149, St. Louis, MO) at room temperature for 10 min. Afterwards, endoproteinase Glu-C (catalog number 90054, Thermo Fisher Scientific, Waltham, MA) was added and the mixture was incubated at 37 °C overnight. The supernatant was collected and the digestion was quenched by addition of a 0.5% formic acid aqueous solution. After incubation at 37 °C for 10 min, the supernatant was collected. The gel pieces were dried by incubation with acetonitrile at 37 °C for 15 min and the supernatant was collected. The combined supernatant was dried in the SpeedVac. A series of calibration curve standards of varying mutant/wild-type (WT) cTnT ratios (0.05, 0.1, 0.2 and 1), but of a fixed total concentration of 20 μM cTnT, was reductively alkylated with DTT and IAA with the same procedure as the in-gel digestion. After mixing with Glu-C and incubation at 37 °C overnight, the digestion reaction was quenched by addition of a 0.5% formic acid aqueous solution. The mixture was then dried in the SpeedVac.

The dried peptides mixture was dissolved in a 0.1% formic acid aqueous solution and the mixture was injected to an Easy Nano-Liquid Chromatography (nLC) II system (Thermo Fisher Scientific, Waltham, MA) equipped with a 75 μm × 10 cm C18AQ analytical column (catalog # SC003, Thermo Fisher Scientific, Waltham, MA) and a 100 μm × 2 cm trap column (catalog # SC001, Thermo Fisher Scientific, Waltham, MA). Mobile phase composition is as follows – A: H2O with 0.1% formic acid; B: acetonitrile with 0.1% formic acid. The gradient profile is linear from 1% B to 35% B in 90 min at 0.3 μl/min. The nLC was online coupled with a Hybrid Velos LTQ-Orbitrap Mass Spectrometer (Thermo Fisher Scientific, Waltham, MA). Eluates from nLC were electrospray-ionized with a 2.2 kV spray voltage. For the initial peptide identification, precursor full mass scans (m/z 350–2000, mass resolution of 60,000 at m/z 400, automatic gain control – AGC target 1 × 106 ions and maximum ionization time 500 ms) were followed by data-dependent collisionalinduced-dissociation (CID) MS2 of the top 9 most abundant precursor ions (AGC target 1 × 105 ions, maximum ionization time 100 ms, isolation window of 2 m/z, normalized collision energy NCE 35 and dynamic exclusion of 60 s). For quantification of percentage of mutant proteins, targeted FT scan (m/z 390–440, mass resolution of 60,000 at m/z 400, AGC target 1 × 106 ions, maximum ionization time 200 ms) were carried out in triplicates for each gel band samples and calibration curve standards.For initial peptide identification, the acquired Xcalibur. raw files were analyzed by Proteome Discoverer 1.4 with Sequest HT (Thermo Fisher Scientific) against a modified human proteome database with troponin T R278C mutant sequence. Variable modification of methionine oxidation and cysteine carboxymethylation were included. Mass tolerance was set with a precursor mass error of less than 5 ppm and MS2 fragment ion mass error of less than 0.8 Da. Peptides with sequence of NQKVSKTRGKAKVTGRWK (TnT WT 271–288, [M + 5H]5+ m/z 415.2498) and NQKVSKTCGKAKVTGRWK (TnT R278C MT 271–288, C278 is carboxylated, [M + 5H]5+ m/z 416.0357) were identified as the R/C278 containing peptides with the highest S/N best suited for quantification. For quantification, the acquired Xcalibur.raw files with the targeted FT scan of m/z 390–440 were manually analyzed in the Xcalibur Qual Browser. Since the retention time (RT) of WT or mutant TnT 271–288 peptides overlaps, the MS signal over the RT with those MS (WT: m/z 415.2498; mutant: m/z 416.0357) signals were averaged and the ratio of mutant/WT MS signals calculated. A calibration curve was established with the above mentioned mutant/WT MS signals ratio and the actual mutant/WT concentration ratio. A linear regression calibration curve was generated with R2 of 0.9964.

### 2.7. RNA sequencing

RNA was isolated using ISOLATE II RNA Mini Kit (Bioline) according to the manufacturer’s instructions with minor adjustments (10 min digestion using 20 μg proteinase K and a subsequent washing step using 100% ethanol were added after the lysis step). Sample quality and quantity was assessed using the 2100 Bioanalyzer with a RNA 6000 Pico Kit (Agilent), and Qubit Flourometer with a HS RNA Assay (Thermo Fisher). After selecting the polyadenylated fraction of RNA, libraries were prepared using the NEXTflexTM Rapid RNA-seq Kit (Bioo Scientific). Libraries were sequenced on the Nextseq500 Illumina platform, producing 75 bp long single end reads. Reads were aligned to the human reference genome GRCh37 using STAR v2.4.2a [23]. Picard’s AddOr-ReplaceReadGroups v1.98 (http://broadinstitute.github.io/picard/) was used to add read groups to the BAM files, which were sorted with Sambamba v0.4.5 [24] and transcript abundances were quantified with HTSeq-count v0.6.1p1 [25] using the union mode. Subsequently, reads per kilobase million reads sequenced (RPKMs) were calculated with edgeR’s RPKM function [26]. The secondary structure of mRNAs were predicted using the RNAfold software, with the minimum free energy (MFE) and partition function option selected (URL: http://rna.tbi.univie.ac.at/cgi-bin/RNAWebSuite/RNAfold.cgi) [27]. WT or mutant full-length human *TNNT2* mRNA sequence corresponding to the adult-expressed isoform of cTnT (NCBI Reference Sequence: NM_001276347.2) was used for the prediction.

### 2.8. Immunofluorescence

Slides were thawed at RT for 20 min inside a closed a box. Tissue sections were washed with PBS-T, permeabilized with 0.25% PBS-Triton and blocked with 1% BSA and 10% donkey serum for 30 min. Primary antibodies for α-actinin (ACTN2, 14221–1-AP, Proteintech, dilution 1:100) and cTnT (TNNT2 [1C11], ab8295, Abcam, dilution 1:250) were incubated overnight at 4 °C. Afterwards tissue sections were washed and incubated with suitable secondary Alexa fluor antibodies for 30 min. The sections were washed in PBS and mounted with Mowiol. Images where acquired with a Nikon A1 confocal microscope and analysis and quantifications were performed with FIJI software.

### 2.9. Statistics

Graphpad Prism v8 software was used for statistical analysis. Data were statistically analyzed using unpaired *t*-test, one-way ANOVA with Dunnett’s or Tukey’s multiple comparisons post hoc test or 2way-ANOVA when appropriate. All values are shown as mean ± standard error of the mean. A *p*-value ≤0.05 was considered as significantly different.

## 3. Results

### 3.1. Lower thin filament-binding affinity of mutant compared to WT troponin complex

We performed troponin exchange in permeabilized human cardiomyocytes to test the direct effect of mutation location and mutant protein dose on myofilament function. Therefore we used the HCM-causing troponin T mutations I79N, R94C and R278C. To analyze their ability to incorporate into the myofilaments and to determine the required complex concentration for a low, intermediate and high protein dose, we exchanged endogenous troponin with different concentrations of recombinant WT and mutant troponin complex. All complexes incorporated to a similar degree (Fig. 1A, C), with a maximum incorporation (Ymax) of 47% for WT, 49% for I79N, 45% for R94C and 50% for R278C. Dose dependency of incorporation appeared to be slightly different for the different complexes. WT and I79N showed almost maximal incorporation at very low concentrations, while R94C and R278C reached the maximal incorporation at higher concentrations (Fig. 1A), which is also illustrated by the difference in k representing the rate constant (k_WT_ = 15.0, k_I79N_ = 22.2, k_R94C_ = 6.2 and k_R278C_ = 3.1) (Fig. 1C). However, the variation is too large to assess whether the complexes exchange differently at low and intermediate dose. Additionally, we performed a co-sedimentation assay with the three complexes to determine their binding affinity to isolated thin filaments. While we observed reduced binding affinity of all three mutants compared to WT, no differences were observed in thin filament binding affinity between the three mutants (Fig. 1D, E, Table S1).

### 3.2. Mutation location determines degree of myofilament dysfunction

**Fig. 2.**
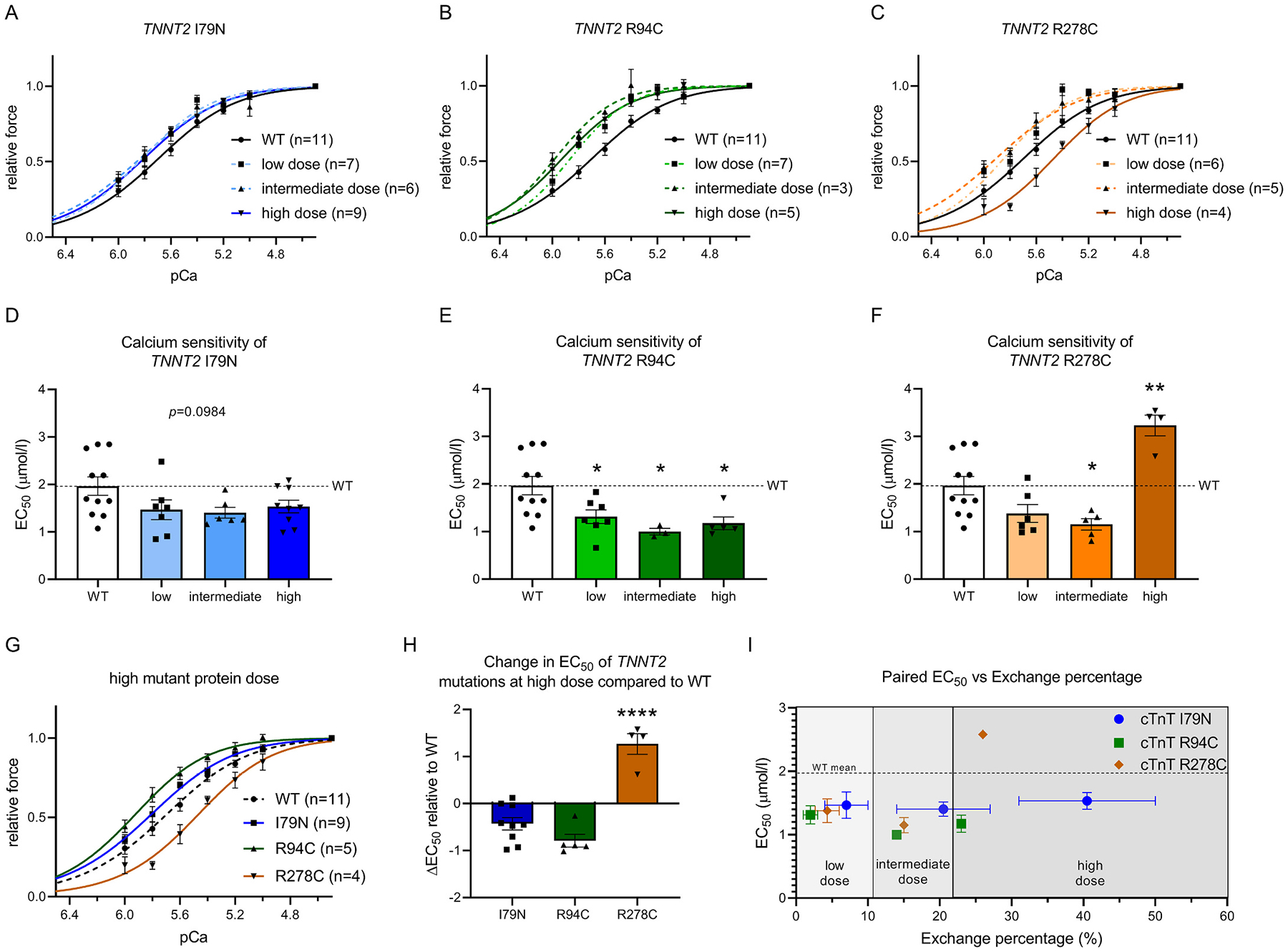
(A-C) Force-calcium relations after exchange with cTnT I79N (A), R94C (B) and R278C (C) complex at low, intermediate and high dose. (D-F) EC_50_ values after exchange with I79N (D), R94C (E) and R278C (F) at low, intermediate and high mutant protein dose. (G) Comparison of force-calcium relation of the different mutants at high dose. (H) Change in EC_50_ (ΔEC_50_) of mutants at high dose compared to WT. (I) Relation of EC_50_ and exchange percentage defined in the same exchange experiments. In (I) n(I79N) = 2/2/2, n(R94C) = 2/1/1, R278C = 3/1/1 for low/intermediate/high dose, respectively. *p < 0.05, **p < 0.01 compared to WT in (D-F); ****p < 0.0001 compared to I79N and R94C in H, one-way ANOVA with Dunnett’s (D-F) or Tukey’s (H) multiple comparisons test.

Based on the obtained percentage of exchange at different concentrations of recombinant troponin complex we selected 0.01 mg/ml, 0.1 mg/ml and 1.5 mg/ml for subsequent functional experiments, which are indicated as low, intermediate and high dose, respectively. Exchange of cTn complex containing the *TNNT2* mutants were compared to cells exchanged with cTn complex containing WT recombinant cTnT (1.5 mg/ml). To avoid that PKA-mediated cTnI phosphorylation differences may mask mutant-related changes in myofilament function, all functional measurements were performed in troponin-exchanged cells after treatment with exogenous PKA. A time-dependent PKA phosphorylation assay of the isolated recombinant complexes confirmed that the cTnI phosphorylation level of the recombinant complex after 40 min of PKA incubation is comparable to that of non-failing donors (Fig. S1). There was no difference in PKA’s ability to phosphorylate the different troponin complexes (Fig. S1E). Moreover, to further minimalize an interfering effect of myofilament protein phosphorylation background, exchange with all 4 troponin complexes (WT and 3 mutants) were performed in the same human sample (sample 2.114). Interestingly, we did not see a mutant protein dose effect for the I79N and R94C mutations (Fig. 2A, B, D, E; Table S2). For the R94C mutation, a significant increase in Ca^2+^-sensitivity was seen at low dose of mutant protein, which did not increase further when increasing the dose (Fig. 2E), indicating that the maximal increase in myofilament Ca^2+^-sensitivity was already achieved at low dose. A similar pattern with a dose-independent trend to increased Ca^2+^-sensitivity (not significant) was observed for I79N (Fig. 2D). The magnitude of the increase in Ca^2+^-sensitivity is determined by the mutation location. We found that R94C increases Ca^2+^-sensitivity to a larger extent than I79N, depicted by the larger curve shift of R94C compared to I79N (Fig. 2G).

Interestingly, we observed strikingly different results for the R278C complex. While the low and intermediate dose led to an increase in Ca^2+^-sensitivity to a similar level as R94C, we observed significantly decreased Ca^2+^-sensitivity for the high dose (Fig. 2C, F; Table S2). This is also demonstrated by the large difference in EC_50_ at low and high dose (Fig. 2F). In several exchange experiments we were able to determine the exchange efficiency in the remaining cell suspension after functional measurements of single cardiomyocytes. In Fig. 2I we plotted myofilament Ca^2+^-sensitivity data relative to the troponin exchange efficiency obtained in experiments in which we collected both data sets. This figure illustrates the mutation location-specific effects on EC_50_.

**Fig. 3.**
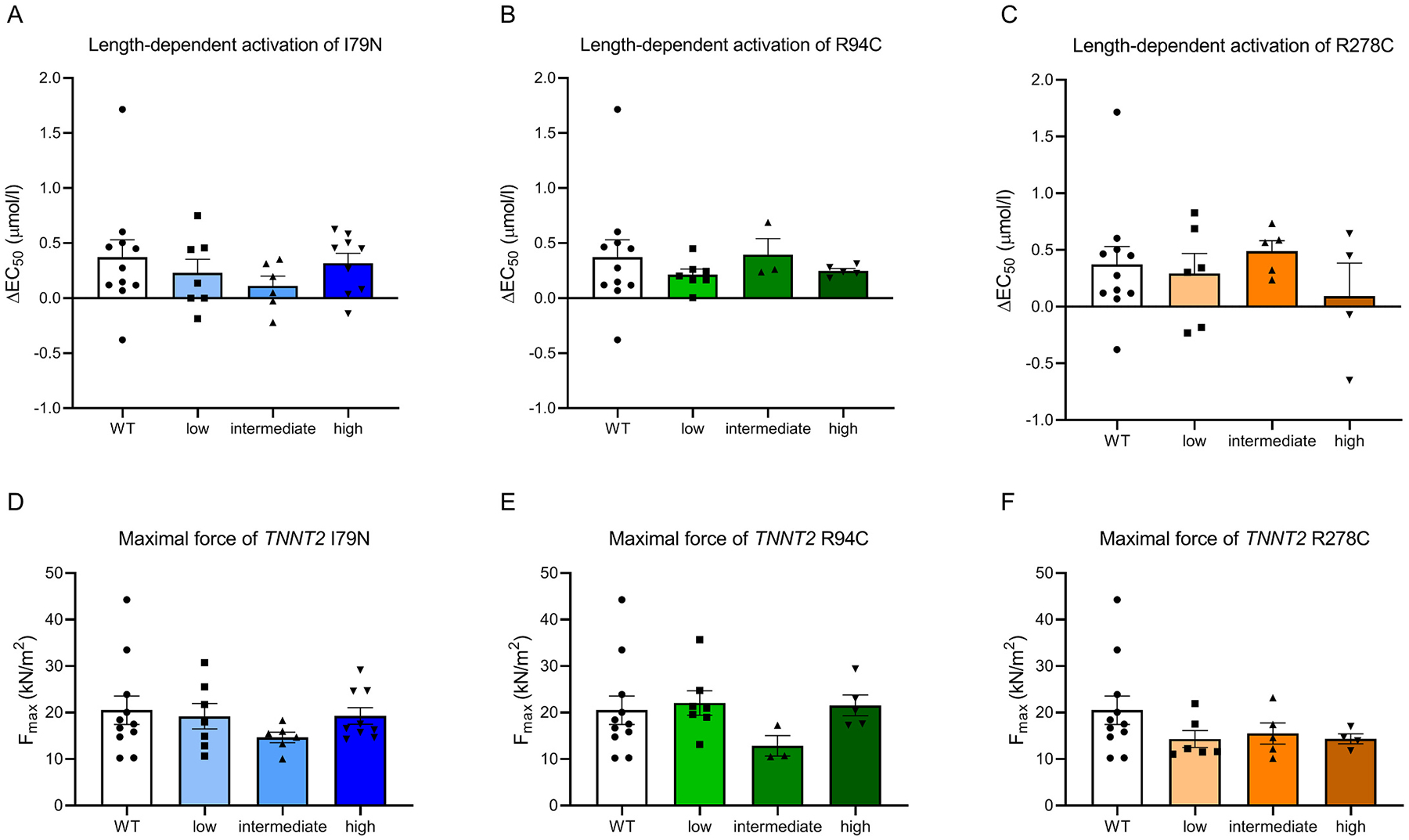
(A), (B) and (C) show length-dependent activation after exchange with cTnT I79N, R94C and R278, respectively. (D), (E) and (F) show maximal force development after exchange with cTnT I79N, R94C and R278, respectively. Low, intermediate and high mutant protein dose are depicted for each mutation compared to WT. n(WT) = 11, n(I79N) = 7/6/9, n(R94C) = 7/3/5, R278C = 6/5/4 for low/intermediate/high dose, respectively.

LDA, the cellular analogue for the Frank-Starling mechanism of the heart, was assessed for all three mutations at low, intermediate and high dose of mutant protein (Fig. 3A–C, Table S3). The variability between cells was large and we did not observe differences in LDA for all three mutations compared to cardiomyocytes exchanged with wild-type troponin complex. Maximal force did not differ between the different mutations and mutant protein dosages (Fig. 3D–F, Table S4).

### 3.3. Expression of R278C at mRNA and protein level in human patient samples

In patients, the percentage of mutant protein in the cell and its contribution to disease variability is unknown. We collected myocardial tissue from the interventricular septum of three obstructive HCM patients carrying the R278C mutation during myectomy surgery (HCM 173, HCM 175 and HCM 234). In these tissues we performed protein analysis and functional measurements to investigate the variability in patients with the exact same mutation.

**Fig. 4.**
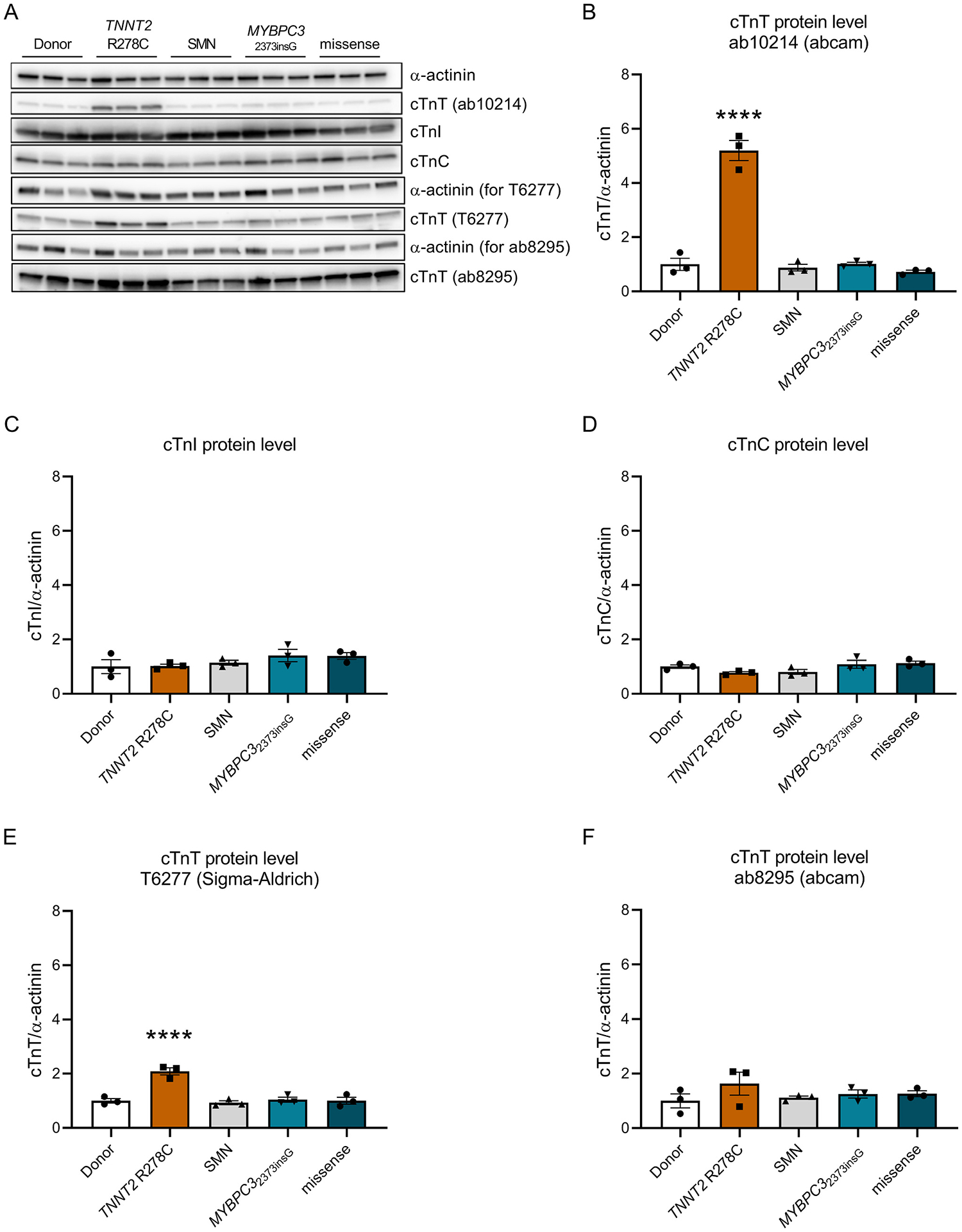
(A) shows representative western blot images of troponin protein levels in the three human samples harboring the *TNNT2* R278C mutation compared to control and other HCM patient samples with the cardiac troponin T (cTnT) antibodies ab10214, T6277 and ab8295. cTnT protein levels with the ab10214 antibody are quantified in (B). (C) shows quantified protein levels of cardiac troponin I (cTnI) and (D) of cardiac troponin C (cTnC). (E) displays quantified cTnT protein levels with the antibody T6277 and (F) with the antibody ab8295. Every dot represents the average of 2 data points per sample in (B), (E) and (F) and the average of 4 data points in (C) and (D). Data is normalized to Donor which is set to 1. ****p < 0.0001 compared to WT, one-way ANOVA with Dunnett’s multiple comparisons test.

**Fig. 5.**
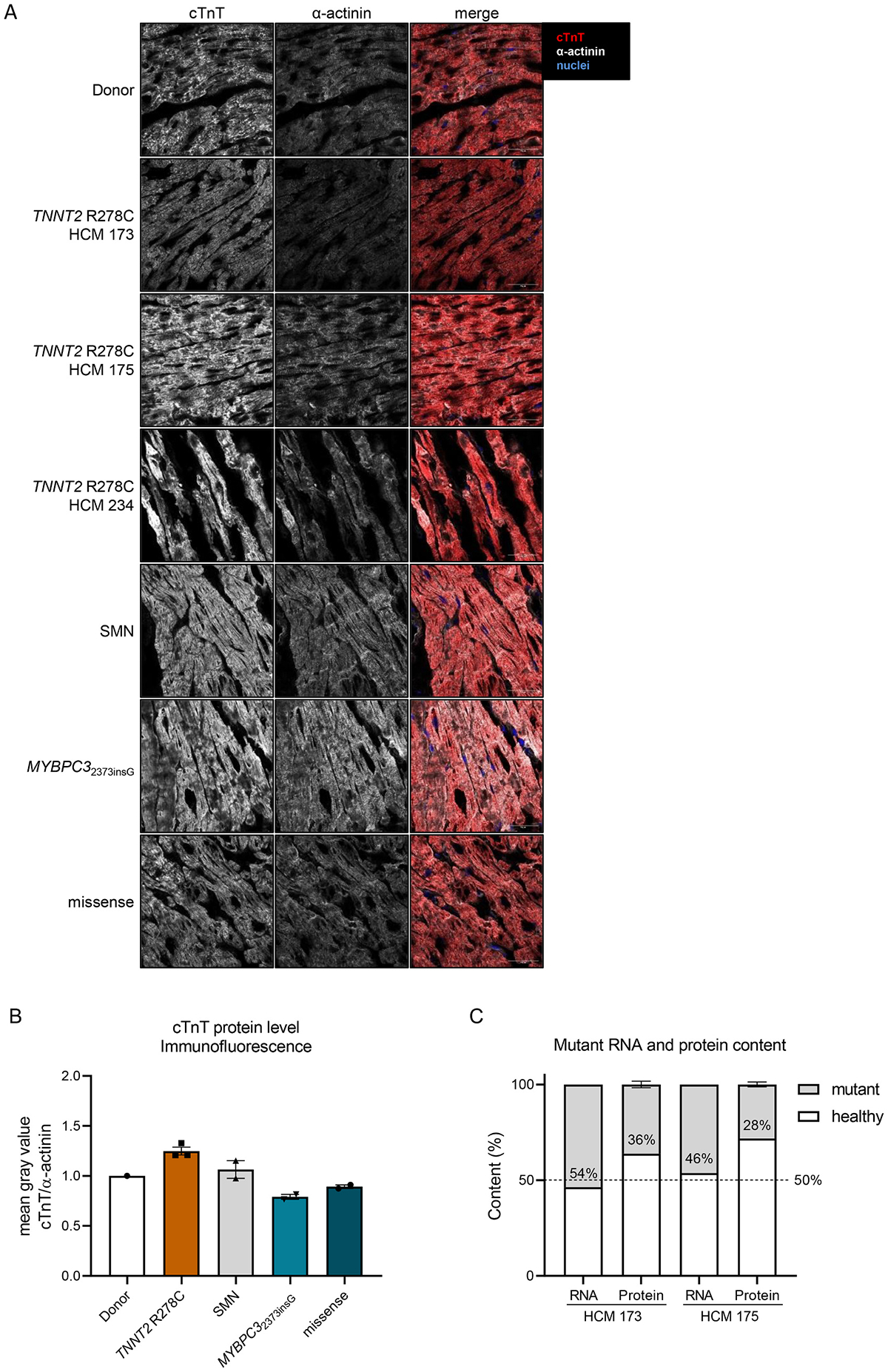
(A) Representative immunofluorescence images of human cardiac tissue stained for cardiac troponin T (cTnT), α-actinin and nuclei. (B) Quantified intensity of cTnT staining normalized to α-actinin staining. Every dot represents one sample and is an average of 3 images. (C) shows the content of mutant cTnT mRNA and protein in the human tissue samples determined by RNA sequencing and mass spectrometry.

Western blot analysis revealed a 5.20 ± 0.37 fold increase in total cTnT protein levels in the three human *TNNT2*-R278C mutant samples compared to donor tissue, sarcomere mutation-negative (SMN) samples, samples with a truncating *MYBPC3* mutation and samples with missense mutations in genes other than *TNNT2*, when analyzed with the ab10214 antibody (abcam) (Fig. 4A, B). Levels of cTnI and cTnC were not changed in the *TNNT2*-R278C samples compared to all other groups (Fig. 4C, D), implying that the presence of mutation R278C in *TNNT2* leads to elevated cTnT levels only. This deviation from the expected troponin stoichiometry of 1:1:1 for cTnT, cTnI and cTnC would be an unexpected unique finding. Therefore, we also determined the cTnT protein levels with the T6277 (Sigma) and the ab8295 (abcam) antibodies to validate the results. Interestingly, the analysis revealed elevated levels of cTnT but with a smaller fold change of 2.09 ± 0.13 for the T6277 antibody (Fig. 4E) and unchanged levels of cTnT with the ab8295 antibody (Fig. 4F). Because of these divergent results, we determined whether the antibodies have the same affinity to recombinant WT and R278C cTnT protein, or whether the mutation affects antibody binding. To do so, we performed western blot analysis on different amounts of recombinant WT and R278C troponin complex. For the two antibodies ab10214 and T6277 we found an approximately 2-fold increase of the cTnT/cTnI signal for the R278C compared to WT, whereas the ab8295 antibody showed an increased cTnT/cTnI signal only at low protein concentrations (Fig. S2A–B). This indicates that the R278C mutation influences antibody binding of all three antibodies, in a possibly protein concentration-dependent manner. We obtained 3 more human *TNNT2*-R278C samples from an Italian patient cohort and compared all available *TNNT2*-R278C samples to two patient samples with other *TNNT*2 mutations (Gln272* and K280N) and could show that the increased cTnT signal is specific for tissue samples harboring the R278C mutation (Fig. S2C). Based on these western blot analyses we cannot make a confident statement about troponin stoichiometry in the *TNNT2* R278C samples. Consequently, we performed immunofluorescence (IF) for cTnT and α-actinin on a selection of the human samples (3 *TNNT2* R278C, 1 Donor, 2 SMN, 2 *MYBPC3*2373insG and 2 missense mutation samples) to determine cTnT protein levels with a different method. Due to the poor performance of the ab10214 and T6277 antibodies in IF, we only used the ab8295 (abcam) antibody for the stainings. In contrast to the western blot analysis of the ab10214 and T6277 antibodies but in line with the results from the ab8295 antibody, IF did not show a significant difference in cTnT protein levels compared to the non-failing donor sample (*p* = 0.0777, Fig. 5A–B). Overall, our data show that the R278C mutation alters cTnT antibody-binding, and cannot be used to determine cTnT protein levels.

We further performed RNA sequencing and mass spectrometry on two HCM tissue samples with the R278C mutation to assess the percentage of the mutant cTnT-R278C mRNA and protein levels in the samples. While *TNNT2*-R278C mRNA levels were close to 50%, the levels of mutant protein were 36% in HCM 173 and 28% in HCM 175 (please note that we were not able to define levels in HCM 234 upon several attempts) (Fig. 5C and Fig. S3). This supports the results from the IF analysis that do not show elevated total cTnT levels in the *TNNT2*-R278C tissues.

### 3.4. R278C mutant human samples differ on a functional level

**Fig. 6.**
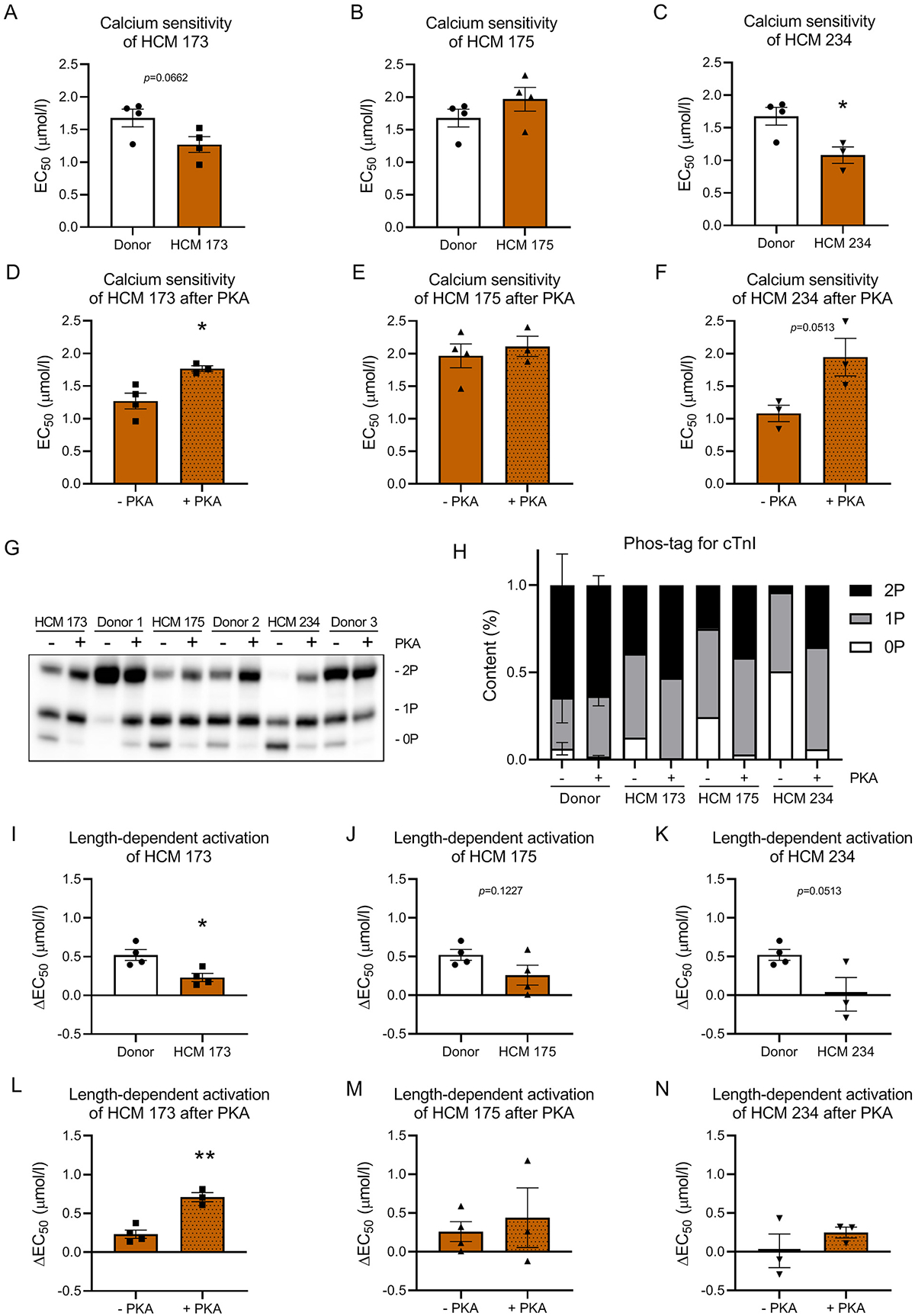
(A-C) show myofilament Ca^2+^-sensitivity measured as EC_50_ for the human samples HCM 173, HCM 175 and HCM 234 carrying the *TNNT2* R278C mutation compared to donor tissue. (D-F) show Ca^2+^-sensitivity measured as EC_50_ for the human samples HCM 173, HCM 175 and HCM 234 carrying the *TNNT2* R278C mutation before and after normalizing cTnI phosphorylation by incubation with protein kinase A (PKA). (G) and (H) display the phosphorylation status of cardiac troponin I determined by phos-tag gel analysis before and after incubation with PKA. 0P represents non-phosphorylated cTnI, 1P single-phosphorylated cTnI and 2P double-phosphorylated cTnI. (G) shows the phos-tag gel image for the quantified data in (H). (I-K) Length-dependent activation of the human samples HCM 173, HCM 175 and HCM 234 compared to donor. (L-N) Length-dependent activation of the human samples HCM 173, HCM 175 and HCM 234 before and after PKA treatment. *p < 0.05, **p < 0.01, unpaired *t*-test. n(Donor) = 4, n(HCM 173) = 4/3, n(HCM 175) = 4/3, R278C = 3/3 for with/without PKA, respectively.

We compared myofilament function of the three patient samples in isolated membrane-permeabilized cardiomyocytes. While the difference in percentage of mutant protein was only slightly different between HCM 173 (36%) and HCM 175 (28%), the direction of the change in Ca^2+^-sensitivity compared to non-failing donors was opposite in these samples, showing a trend to an increase and a decrease, respectively (Figs. 6A–B, Table S5). HCM 234 shows a significant increase in Ca^2+^-sensitivity compared to donor samples (Fig. 6C, Table S5). Analysis of cTnI phosphorylation revealed reduced levels of phosphorylation in all three patient samples compared to donors, shown by lower levels of the highly phosphorylated 2P band and increased levels of the 1P and 0P bands (Fig. 6G–H). The lowest level of cTnI phosphorylation was observed in HCM 234. Since it is well-known that reduced cTnI phosphorylation increases myofilament Ca^2+^-sensitivity [16], we repeated the functional measurements after incubation with PKA to increase cTnI phosphorylation in HCM samples to levels observed in non-failing donor samples. First of all we determined with a phosphorylation assay if cTnI is still PKA responsive in these patients. As seen in Fig. 6G–H, cTnI phosphorylation increases after treatment with PKA in the *TNNT2* mutant samples, indicating that PKA-mediated phosphorylation of cTnI is not hampered by the presence of the *TNNT2* mutation. Accordingly, PKA treatment of isolated cardiomyocytes decreases myofilament calcium sensitivity significantly in HCM 173 and with a strong trend in HCM 234 (Fig. 6D–F, Table S5). In line with these results, we observed a blunted LDA in all three patient samples compared to donor, but detected a statistically significant difference only for HCM 173 (Fig. 6I–K, Table S6). LDA was enhanced only in HCM 173 after PKA-mediated phosphorylation of cTnI (Fig. 6L–N, Table S6).

Overall, our data suggest that perturbations in myofilament function in samples harboring the R278C mutation can be corrected to values that are seen in non-failing samples by PKA treatment.

## 4. Discussion

In this study we investigated the mutation location and mutant protein dose effect of three HCM-causing *TNNT2* mutations. We proposed that the mutation location and mutant protein dose are potential disease modifiers via specific changes in myofilament function. While our exchange experiments show a more important role for the mutation location than for the mutant protein dose for mutations I79N and R94C, the functional consequences of R278C are highly dose-dependent.

### 4.1. Mutant protein dose effect is mutation-specific

HCM mutations in thin filament proteins such as cTnT have been associated with an increase in myofilament Ca^2+^-sensitivity [28,29] and blunted length-dependent myofilament activation [28]. Impaired LDA was normalized to values in non-failing donor cardiomyocytes by reducing the percentage of mutant cTnT to 22% [28]. In the present study, we show that a relatively low dose of mutant cTnT protein is sufficient to increase myofilament Ca^2+^-sensitivity, which was siginificant for R94C, though the magnitude is dependent on mutation location (Fig. 2).

At low, intermediate and high dose of mutant protein we did not observe differences in LDA (Fig. 3). R94C led to an increase in myofilament Ca^2+^-sensitivity independent of dose and also I79N showed a strong trend to increased dose-independent myofilament Ca^2+^-sensitivity, with R94C having an even stronger effect than I79N. The Ca^2+^-sensitizing effect of I79N has been described previously [12,29,30], and also for R94C a Ca^2+^-sensitizing effect and hypercontractility has been predicted [31]. In our study, a very low dose of mutant protein results already in the maximal shift in myofilament Ca^2+^-sensitivity (Fig. 2I). In contrast to our study in human cardiomyocytes, the R92Q mutation showed a dose-dependent effect on atrial mass, hypertrophic markers and structural abnormalities in a Tg mouse model [10]. Unlike the direct mutation effects that we studied in single permeabilized cardiomyocyte, the dose-dependent changes in cardiac remodeling described in the Tg-mice may represent secondary changes and may not be solely due to the direct mutation dose effect.

Several studies in animal models and patients have also described that individuals with two disease-causing mutations show earlier disease onset, more severe hypertrophy and a higher risk of sudden cardiac death [32–34]. Double mutations in the same gene, dependent on their location, might have an additive effect as described for two *MYBPC3* mutations [35]. Compound heterozygous or homozygous *MYBPC3* mutations have also been shown to cause severe cardiomyopathy which was lethal in the first few weeks after birth [36,37]. An additive mutation dose effect has also been shown for double heterozygous mutations in two different genes like *MYH7* and *CSRP3* [38]. These studies indicate that the mutation dose effect might not always be in the dose of one gene, but rather in the combination of mutations in different genes that increase the cellular burden resulting in a more severe clinical outcome.

In contrast to the findings with the I79N and R94C mutations, R278C shows a trend to increased Ca^2+^-sensitivity at low and a significant increase at intermediate dose, while a high dose significantly decreased Ca^2+^-sensitivity (Fig. 2C, F, I). Conflicting results regarding this mutation have been reported in literature, showing either a Ca^2+^-sensitization or no effect on Ca^2+^-sensitivity [13,29]. Considering the findings of our study, the different reported effects on myofilament function could be explained by differences in mutant protein dose. The different behavior of the N-terminal *TNNT2* mutations (I79N and R94C) and the C-terminal mutation R278C may also be explained by their interactions with other thin filament proteins, e.g. tropomyosin and cTnC as calcium binding to cTnC triggers conformational changes in troponin and tropomyosin that subsequently allow interactions between actin and myosin heads. The stoichiometry of 7:1:1 for actin, tropomyosin and troponin underlies the tight regulation of calcium-induced cross-bridge formation and myofilament force development. Molecular models based on crystal structures showed that the N-terminal region of cTnT (residues 87–150) is extremely elongated and interacts with both tropomyosin and 3 out of 7 actin subunits [39], and stabilizes tropomyosin-troponin binding to actin. As the cTnT N-terminus bridges over two tropomyosin strands [39], small amounts of mutant *TNNT2* in this region may already exert a maximal effect on myofilament Ca^2+^-sensitivity via propagation of the calcium-binding signal over the thin actin-filament. Furthermore, it has recently been shown that the C-terminus of cTnT, at which the R278C mutation is located, can directly interact with cTnC [19]. This cTnT-cTnC interaction may be altered by the presence of mutant R278C in a dose-dependent manner, and thereby differentially alter cTnC sensitivity to calcium.

Interestingly, a divergent effect of a *TNNT2* mutation has been reported before. The introduction of the dilated cardiomyopathy (DCM)-causing *TNNT2* mutation ΔK210 in reconstituted thin filaments led to decreased Ca^2+^-sensitivity when using WT and ΔK210 cTnT in a 50:50 ratio, whereas it resulted in increased Ca^2+^-sensitivity when using 100% ΔK210 [40]. These findings indicate that dose-dependency of the functional defect might not be a common disease mechanism for all *TNNT2* mutations but might depend on mutation location. Moreover, based on the elegant work by Davis and colleagues, who build proof for tension-mediated cardiomyocyte remodeling [41], the R278C mutation may trigger concentric remodeling (i.e. HCM phenotype) at low dose, and eccentric remodeling (i.e. DCM phenotype) at high protein dose.

### 4.2. Mutation location determines myofilament alterations

The differences between the mutations in this study could be due to mutation-specific changes in protein properties like structure or charge, that have differential effects on protein function by e.g. influencing their interaction with binding partners. In a study with *MYH7* mutations, differences in disease severity have been proposed to depend on the mutation location due to their effect on different protein domains that might be of more or less importance for proper protein function [42]. Some of these mutations could be associated with either mild or severe disease phenotype [43,44]. Also for *ACTC1* it has been described that the subdomain location of the mutation determines the alterations in protein properties. Mutations in one subdomain predominantly affected protein stability or polymerization, whereas mutations in other sub-domains caused alterations in protein-protein interactions [45]. Vang et al. have shown in in vitro experiments that different HCM- and DCM-causing mutations in *ACTC1* impair protein folding by the TRiC chaperonin complex resulting in inefficient incorporation into the myofilament and aggregation of actin [46]. A similar mechanism could potentially explain the lower thin filament binding affinity of the *TNNT2* mutant complexes compared to WT (Fig. 1D). Pavadai et al. describe that the R94 residue, together with other charged residues, forms stable salt bridges that border hydrophobic patches leading to a very tight interaction of the cTnT fragment with residues 89–151 and tropomyosin [47]. Having an uncharged cysteine (C) instead of the positively charged arginine (R) at residue 94 may weaken the interaction of cTnT with tropomyosin and explain the reduced binding affinity of R94C to the thin filament. A similar mechanism may be true for the R278C mutation, which shows the same amino acid change. Accordingly, also the change from a non-polar isoleucine (I) to a polar asparagine (N) for the I79N mutation may potentially alter the interaction of cTnT with tropomyosin.

Differences in exchangeability into porcine skinned fibers of different mutant cTnT proteins was shown in a previous study, in which R92W, R94L or R130C showed reduced exchangeability compared to WT and other cTnT mutant proteins (A104V, E163R, S179F, E244D) [15]. This is in line with findings by Palm et al., who have shown that mutations within the residues 92–110 alter the interaction of cTnT with tropomyosin [48]. Furthermore, studies in Tg mice showed less efficient incorporation of mutant compared to WT cTnT. Tg-I79N lines expressed 52% and 35% Tg-cTnT compared to 71% Tg-cTnT in the Tg-WT line [12]. Similar results have been reported for the Tg-cTnI mouse lines R145G and R145W that only showed 36% and 11% of mutant Tg-cTnI compared to 66% of Tg-cTnI in the Tg-WT line [49,50].

Overall, our study strengthens the notion that the degree of myofilament dysfunction depends on mutation location, and that mutant cTnT protein reduces thin filament binding affinity of the troponin complex.

### 4.3. Altered antibody binding affinity to cTnT-R278C

Troponins function as part of a complex, which is closely linked with tropomyosin. It has been shown that mutations in one of the troponin units can affect the composition of the entire troponin complex. In a study of different DCM-causing troponin mutations it has been shown for a truncating mutation in *TNNI3* and an amino acid deletion in *TNNT2* that the stoichiometry of the troponin complex was altered. In these DCM patient samples the mutation also affected the protein levels of its binding partners [51]. Although part of our western blot data suggests elevated cTnT levels in the *TNNT2*-R278C samples, we have shown in Fig. S2 that all cTnT antibodies show increased binding specifically to the mutant R278C compared to WT protein. Although the cTnT antibody epitope is more than 100 amino acids away from the mutation site, the amino acid change in the mutant protein is influencing antibody binding in western blot analysis. This, in combination with the ab8295 western blot and IF results, which did not show higher cTnT protein levels compared to donor (Fig. 5), suggest that there is no change in troponin stoichiometry in the HCM patient samples carrying the *TNNT2*-R278C mutation.

This study shows that special care has to be taken when analyzing mutant proteins as antibody binding can be affected even under denaturing conditions.

Our RNA analyses showed a ~50/50% ratio of WT and mutant mRNA in the *TNNT2* R278C cardiac patient samples, while mass spectrometry revealed that the amount of mutant protein was less than 50%. This finding is in line with our in vitro data that show reduced binding of the mutant protein to the thin filament compared to WT and may explain the lower percentage of mutant protein in the human tissue. The predicted mRNA structures for WT and R278C mRNA differ, suggesting that R278C mRNA may indeed have different characteristics than WT mRNA (Fig. S4), which may affect its translation efficiency, resulting in lower protein levels of cTnT-R278C. Alternatively, it may be speculated that the turnover of WT cTnT protein is reduced, a mechanism which was recently proposed for *MYBPC3* truncating mutations to maintain normal cMyBP-C levels and prevent haploinsufficiency [52]. Future research is warranted to investigate mechanisms underlying protein translation and turnover in order to define the role of both WT and mutant proteins in sarcomere homeostasis.

### 4.4. Differences in myofilament function between human TNNT2 R278C samples do not correspond with mutant protein dose

Interestingly, our human samples with the R278C mutation showed differences in myofilament Ca^2+^-sensitivity, while their clinical characteristics were very similar (Table 1) and samples were all collected at the time of myectomy. HCM 173 showed a strong trend to increased myofilament Ca^2+^-sensitivity and HCM 234 showed a significant increase in myofilament Ca^2+^-sensitivity compared to donor, whereas HCM 175 was not different. Based on the dose-dependent effect observed for the R278C mutation in our exchange experiments, i.e. high myofilament Ca^2+^-sensitivity at low dose, and low myofilament Ca^2+^-sensitivity at high mutant dose, mutant protein levels should be higher in the HCM 175 than in the HCM 173 sample. However, our mass spectrometry data showed a slightly lower mutant protein level in HCM 175 (28%) than in HCM 173 (36%).

A well-known modifier of Ca^2+^-sensitivity is PKA-mediated phosphorylation of cTnI [16]. While the level of cTnI phosphorylation was lower in all three samples compared to non-failing donor tissue, only HCM 173 and HCM 234 displayed increased Ca^2+^-sensitivity. Restoring phosphorylation of cTnI indeed decreased Ca^2+^-sensitivity significantly in HCM 173 and with a strong trend in HCM 234. Although PKA phosphorylation of cTnI is a major regulatory mechanism of myofilament Ca^2+^-sensitivity, there are many more post-translational protein modifications that could potentially alter myofilament function. Also protein kinase C (PKC)-mediated phosphorylation can influence myofilament Ca^2+^-sensitivity and has been shown to decrease it [53]. Therefore, enhanced PKC activity may explain why Ca^2+^-sensitivity is not reduced in HCM 175 while the sample does show reduced phosphorylation of cTnI.

Additionally, myofilament Ca^2+^-sensitivity could be influenced by a range of other factors, that are beyond the scope of this study. We can speculate that the extent of fibrosis, medication and the presence of comorbidities can influence myofilament function by altering post-translational modifications of myofilament proteins.

Overall, our data illustrate that myofilament Ca^2+^-sensitivity measured at the time of myectomy in HCM samples harboring *TNNT2* mutations is highly diverse, and most likely reflect a complicated mix of translational and post-translational protein modifications. The latter is in line with our previous observations in HCM and DCM samples with thin filament gene mutations [28,51], and also matches previously reported conflicting results on the R278C mutation. One study showed dramatically increased Ca^2+^-sensitivity [29], whereas another one described R278C as a rather benign mutation with no effect on Ca^2+^-sensitivity [13].

## 5. Study limitations

This study gives new insight into the effect of mutant protein dose and location of *TNNT2* mutations on human myofilament function. It is a limitation of the study that we only assessed two N-terminal mutations and one C-terminal mutation, which all show differences on a functional level. To make a more general statement on the effect of mutations in certain cTnT domains, additional mutation locations have to be investigated. Furthermore, our study shows that isolated mutation effects are not directly translatable to the human situation, since several other disease modifying factors can influence the impact of the mutant protein on the cell and lead to differences in the functional state of the cardiomyocytes between patients with the same mutation.

## 6. Conclusion

From our exchange experiments we can conclude that mutant protein dose-dependency may be relevant only for certain *TNNT2* mutations. Based on our experiments, this may be true for mutations located in the C-terminal domain that interacts with other troponin subunits. Our studies show that a relatively small dose of mutant protein is sufficient to exert the maximal effect on myofilament Ca^2+^-sensitivity for the I79N and R94C mutation, while the mutation location determines the magnitude of this effect. This could be explained by the interaction of troponin with tropomyosin which is involved in the regulation of cooperativity of thin filament activation. Our study emphasizes that care has to be taken when analyzing mutant proteins as single amino acid changes can alter antibody binding even under denaturing conditions. The ‘classical’ view of the HCM-related myofilament Ca^2+^-sensitization clearly is too simplified, as our study shows that thin filament-based HCM pathology involves additional levels of complexity including sarcomere homeostasis and mutation-specific effects.

## Supplementary Material

SI

## Figures and Tables

**Table 1:** Clinical characteristics of HCM samples.

Sample ID	Age (yrs, at myectomy)	Sex	Genotype	IVS (mm)	LAD (mm)	EDD (mm)	E/A ratio	E/e’ ratio	Diastolic dysfunction (stage)	LVOTO (mmHg)
HCM 173	58	M	*TNNT2* R278C	18	51	40	1.5	15.7	2	74
HCM 175	61	M	*TNNT2* R278C	16	46	50	0.75	12.5	2	31
HCM 234	72	M	*TNNT2* R278C	19	62	38	*NA*	20.8	2 or 3	96
FL 1084	49	M	*TNNT2* R278C & *MYBPC3* T1095M	*NA*	*NA*	*NA*	*NA*	*NA*	*NA*	*NA*
FL 1097	60	F	*TNNT2* R278C	*NA*	*NA*	*NA*	*NA*	*NA*	*NA*	*NA*
FL 10112	73	F	*TNNT2* R278C & *MYBPC3* K814del	*NA*	*NA*	*NA*	*NA*	*NA*	*NA*	*NA*
HCM 132	15	F	*TNNT2* Gln272*	19	37	39	2.33	17.5	3	81
SHB 3166	26	M	*TNNT2* K280N	37	*NA*	*NA*	*NA*	*NA*	*NA*	*NA*
HCM 109	71	F	SMN	22	52	53	1.30	33.6	2	130
HCM 168	46	M	SMN	15	49	41	1.00	20.4	2	92
HCM 222	68	M	SMN	15	45	49	1.39	27.2	2	61
HCM 169	52	M	*MYBPC3* 2373insG	21	45	43	0.87	15.9	1	100
HCM 204	30	M	*MYBPC3* 2373insG	19	37	37	1.14	16.3	2	100
HCM 219	44	F	*MYBPC3* 2373insG	19	48	43	1.5	12.5	3	74
HCM 163	64	F	*TNNI3* R145W	23	46	42	0.52	16.3	1	125
HCM 236	29	M	*MYH7* T1377M	26	52	44	1.83	18.3	2	104
HCM 246	25	M	*MYH7* R663C	28	34	*NA*	*NA*	*NA*	*NA*	38

Clinical characteristics of human HCM samples that were used in this study.

Abbreviations: sarcomere mutation negative (SMN), interventricular septum (IVS), left atrial diameter (LAD), enddiastolic diameter (EDD), left ventricular outflow tract obstruction (LVOTO). FL, samples from Florence; SHB, explanted heart sample from Sydney Heart Bank.

* Indicates a truncation after the amino acid.
