# Supplementary material for: Mutation location of HCM-causing troponin T mutations defines the degree of myofilament dysfunction in human cardiomyocytes
Source: J Mol Cell Cardiol. Author manuscript; Available in PMC 2023 Oct 31. (PMC10616699; doi:10.1016/j.yjmcc.2020.10.006)
Supplement: SI [file NIHMS1937901-supplement-SI.pdf]

Supplemental figures and tables

Title:

Mutation location of HCM-causing troponin T mutations defines the degree of myofilament dysfunction in human cardiomyocytes

Authors:

Maike Schuldt[a], Jamie R. Johnston[b], Huan He[b,c], Roy Huurman[d], Jiayi Pei[e,f], Magdalena Harakalova[e,f], Corrado Poggesi[g], Michelle Michels[d], Diederik WD. Kuster[a], Jose R. Pinto[b], Jolanda van der Velden[a]

Affiliations:

[a]Amsterdam UMC, Vrije Universiteit Amsterdam, Department of Physiology, Amsterdam Cardiovascular Sciences, Amsterdam, the Netherlands

[b]Department of Biomedical Sciences, College of Medicine, Florida State University, Tallahassee, FL, USA

[c]Institute of Molecular Biophysics, Florida State University, Tallahassee, FL, USA

[d]Department of Cardiology, Thorax Center, Erasmus Medical Center, Rotterdam, the Netherlands

[e]Department of Cardiology, Division Heart and Lungs, University Medical Center Utrecht, Utrecht, The Netherlands

[f]Regenerative Medicine Utrecht, University Medical Center Utrecht, Utrecht, The Netherlands

[g]Department of Experimental and Clinical Medicine, University of Florence, Florence, Italy

Corresponding author:
Maike Schuldt, m.schuldt@amsterdamumc.nl

O2 building 11W53, De Boelelaan 1117, 1081HV Amsterdam, the Netherlands

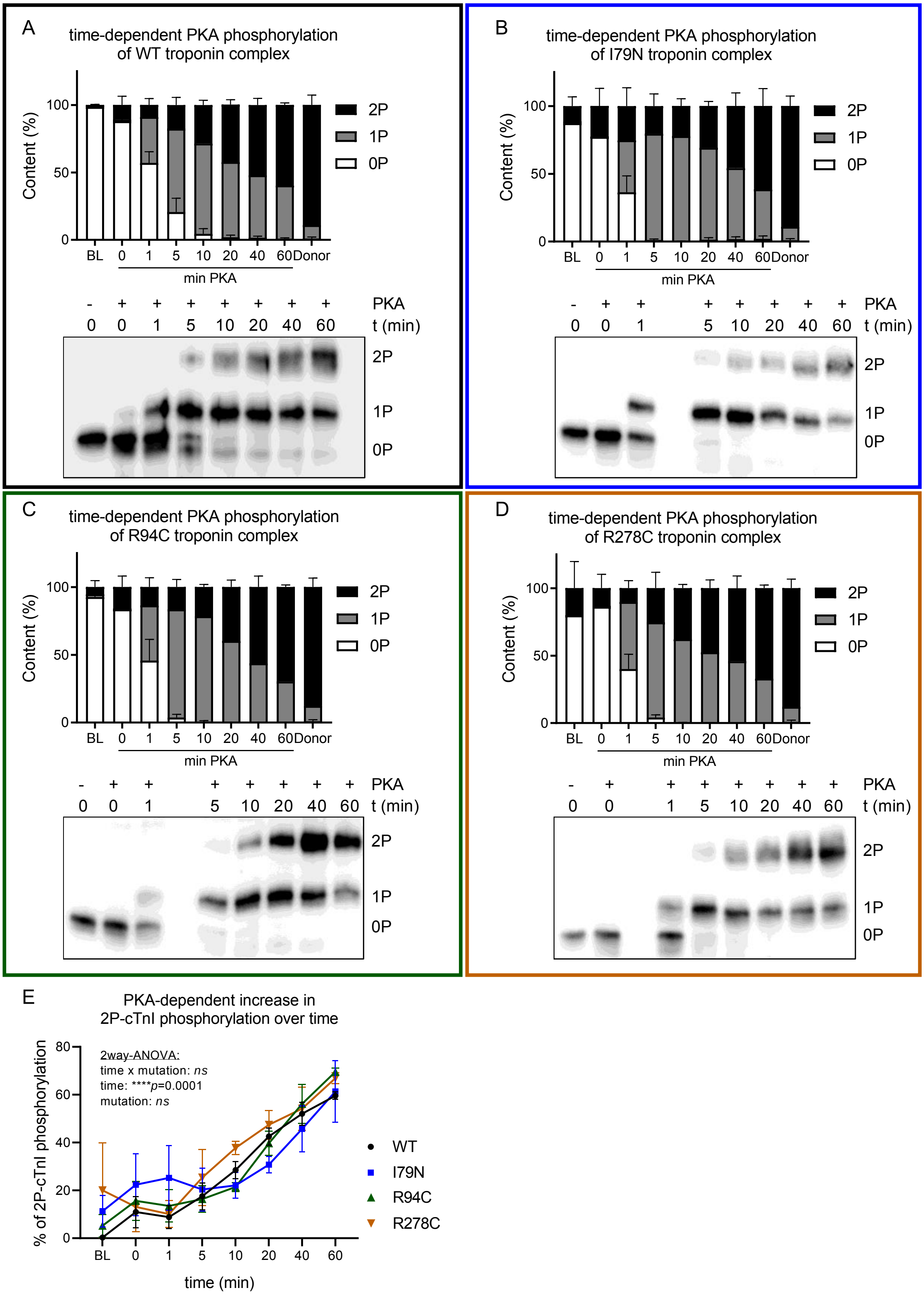

Figure S1. Phosphorylation assay of recombinant troponin complexes. Recombinant troponin complex was incubated with 10 units of protein kinase A (PKA) in a time-dependent manner. Phosphorylation was analyzed using phos-tag gel electrophoresis. Assay of (A) wildtype (WT) troponin complex, (B) *TNNT2* I79N complex, (C) *TNNT2* R94C complex and (D) *TNNT2* R278C complex. (E) PKA-dependent increase in 2P-cTnI phosphorylation from A-D over time for the different complexes, analyzed with 2way-ANOVA. n=3.

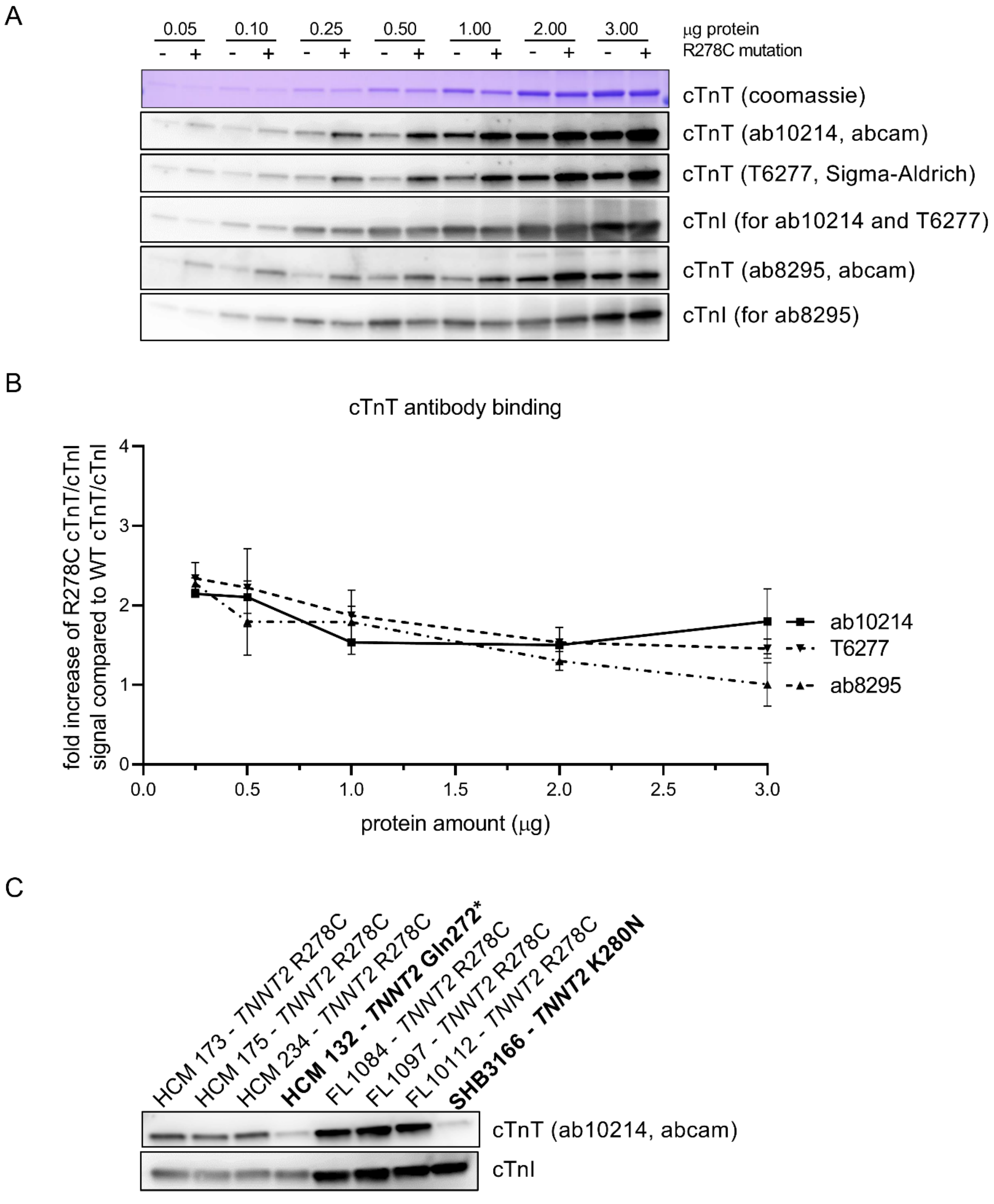

Figure S2. (A) Coomassie staining of a gel showing equal loading of WT and R278C complex at different protein amounts and western blot analysis of cardiac troponin T (cTnT) of different amounts of WT and R278C complex with the cTnT antibodies ab10214, T6277 and ab8295. (B) Quantified western blot data of (A), showing the fold increase of the cTnT/cardiac troponin I (cTnI) signal of the R278C complex compared to WT (only the data points that fall into the linear range of the antibody are shown), n=2. (C) Western blot analysis of six human samples with the *TNNT2*-R278C mutation and two samples with *TNNT2* mutations other than the R278C mutation.

A

B

C

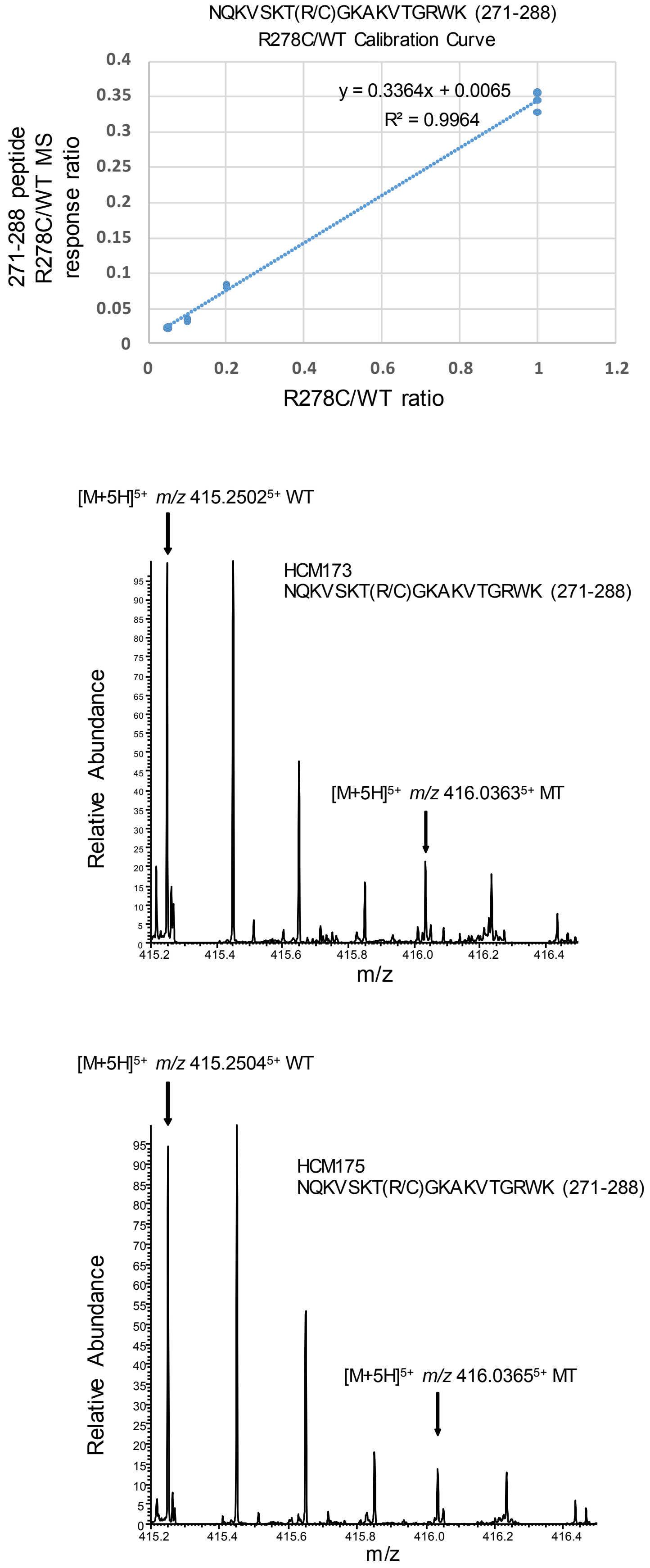

Figure S3. Protein expression of wild-type (WT) and R278C-mutant cTnT by mass spectrometry.
(A) Calibration curve to determine the percentage of R278C-mutant (MT)/WT cTnT. Y axis shows the MS signal ratio of R278C peptide vs WT peptide. X axis shows the actual R278C/WT protein concentration ratio. A linearity regression was achieved with R^2 of 0.9964. (B) MS spectrum of WT and R278C-mutant cTnT peptides in HCM 173. (C) MS spectrum of WT and R278C-mutant cTnT peptides in HCM 175.

A

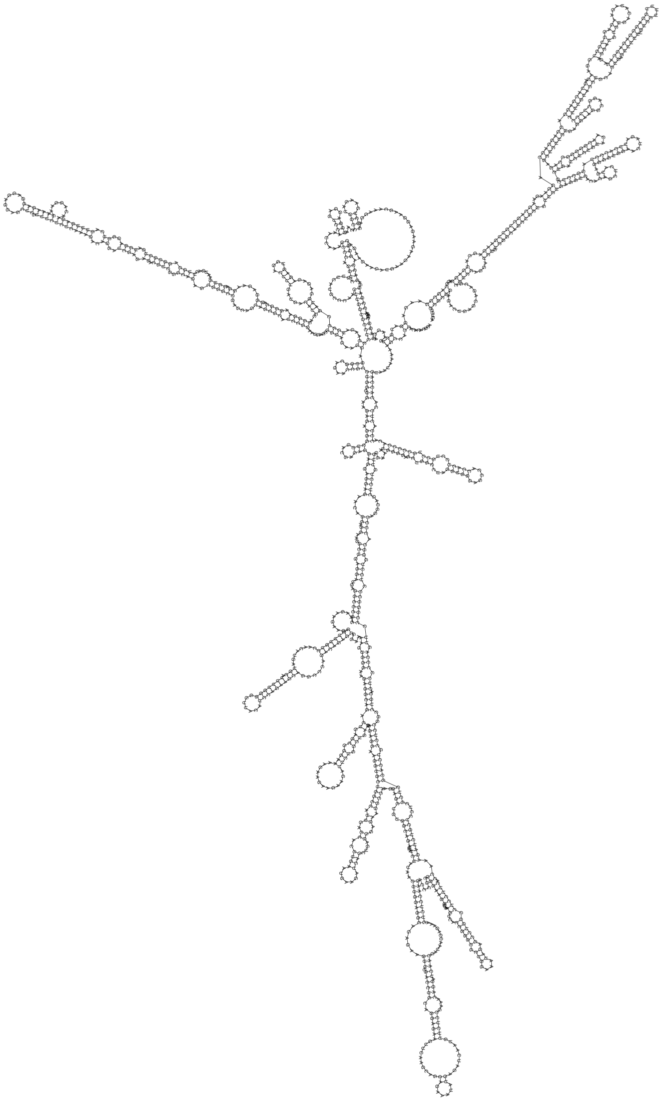

TNNT2 WT mRNA

B

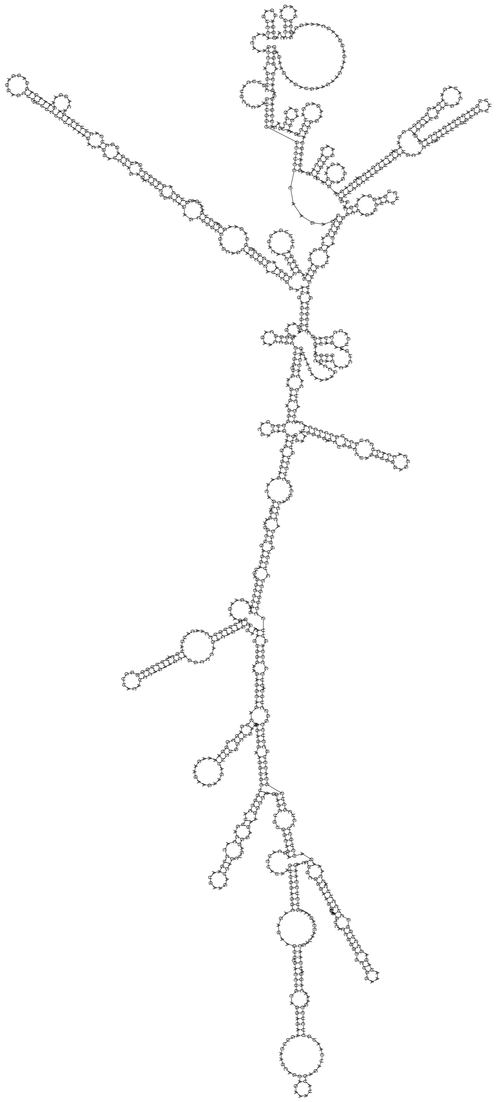

TNNT2 R278C mRNA

Figure S4. Predicted mRNA structure of WT and R278C *TNNT2* mRNA.

Table S1: Summary data of co-sedimentation assay. Data are analyzed with one-way ANOVA and Dunnett's multiple comparisons test.

**sample**	**mean cTnI/Tm band intensity ± SEM**	**n**	***p* value**
cTnT-WT	0.58 ± 0.01	4	
cTnT-I79N	0.43 ± 0.02	4	***0.0014*
cTnT-R94C	0.46 ± 0.02	4	**0.0102*
cTnT-R278C	0.46 ± 0.03	4	**0.0105*

Table S2: Summary data of calcium sensitivity measurements of troponin exchange experiments. Data are analyzed with a one-way ANOVA per mutation and Dunnett's multiple comparisons test.

**sample**	**mean EC_{50} ± SEM**	**n**	***p* value**
cTnT-WT	1.96 ± 0.19	11	
cTnT-I79N low	1.47 ± 0.21	7	0.1376
cTnT-I79N intermediate	1.40 ± 0.11	6	0.1031
cTnT-I79N high	1.53 ± 0.13	9	0.1795
cTnT-R94C low	1.31 ± 0.14	7	**0.0346*
cTnT-R94C intermediate	1.00 ± 0.07	3	**0.0188*
cTnT-R94C high	1.17 ± 0.13	5	**0.0203*
cTnT-R278C low	1.38 ± 0.19	6	0.1037
cTnT-R278C intermediate	1.15 ± 0.12	5	**0.0245*
cTnT-R278C high	3.23 ± 0.22	4	***0.0012*

Table S3: Summary data of length-dependent activation measurements of troponin exchange experiments. Data are analyzed with a one-way ANOVA per mutation and Dunnett's multiple comparisons test.

**sample**	**mean ΔEC_{50} ± SEM**	**n**	***p* value**
cTnT-WT	0.37 ± 0.16	11	
cTnT-I79N low	0.23 ± 0.12	7	0.7944
cTnT-I79N intermediate	0.11 ± 0.09	6	0.4262
cTnT-I79N high	0.32 ± 0.09	9	0.9809
cTnT-R94C low	0.21 ± 0.05	7	0.7382
cTnT-R94C intermediate	0.40 ± 0.15	3	0.9994
cTnT-R94C high	0.25 ± 0.02	5	0.8861
cTnT-R278C low	0.29 ± 0.18	6	0.9785
cTnT-R278C intermediate	0.49 ± 0.09	5	0.9478
cTnT-R278C high	0.09 ± 0.29	4	0.6554

Table S4: Summary data of maximal force measurements of troponin exchange experiments. Data are analyzed with a one-way ANOVA per mutation and Dunnett's multiple comparisons test.

**sample**	**mean F_{max} ± SEM**	**n**	***p* value**
cTnT-WT	20.50 ± 3.07	11	
cTnT-I79N low	19.20 ± 2.73	7	0.9723
cTnT-I79N intermediate	14.64 ± 1.13	6	0.3167
cTnT-I79N high	19.27 ± 1.80	9	0.9711
cTnT-R94C low	22.06 ± 2.63	7	0.9665
cTnT-R94C intermediate	12.84 ± 2.21	3	0.3850
cTnT-R94C high	21.53 ± 2.22	5	0.9925
cTnT-R278C low	14.32 ± 1.81	6	0.2969
cTnT-R278C intermediate	15.50 ± 2.27	5	0.5154
cTnT-R278C high	14.35 ± 1.07	4	0.4126

Table S5: Summary data of calcium sensitivity measurements of human *TNNT2* R278C samples. Baseline data are analyzed with unpaired student's t-test compared to donor, PKA treated samples are analyzed with unpaired student's t-test compared to the corresponding baseline measurement.

**sample**	**mean EC_{50} ± SEM**	**n**	***p* value**
Donor	1.68 ± 0.14	4	
HCM 173	1.27 ± 0.12	4	0.0662
HCM 175	1.97 ± 0.18	4	0.2501
HCM 234	1.08 ± 0.13	3	**0.0270*
HCM 173 + PKA	1.77 ± 0.04	3	**0.0195*
HCM 175 + PKA	2.11 ± 0.15	3	0.5894
HCM 234 + PKA	1.95 ± 0.29	3	0.0513

Table S6: Summary data of length dependent activation measurements of human *TNNT2* R278C samples. Baseline data are analyzed with unpaired student's t-test compared to donor, PKA treated samples are analyzed with unpaired student's t-test compared to the corresponding baseline measurement.

**sample**	**mean ΔEC_{50} ± SEM**	**n**	***p* value**
Donor	0.52 ± 0.07	4	
HCM 173	0.23 ± 0.05	4	**0.0165*
HCM 175	0.26 ± 0.13	4	0.1227
HCM 234	0.01 ± 0.22	3	0.0513
HCM 173 + PKA	0.71 ± 0.06	3	***0.0019*
HCM 175 + PKA	0.44 ± 0.38	3	0.6323
HCM 234 + PKA	0.25 ± 0.07	3	0.3591
